# Supplementary material for: A 22-G or a 25-G Needle: Which One to Use in the Diagnostics of Solid Pancreatic Lesions? A Systematic Review and Meta-Analysis
Source: Cancers (Basel). 2024 Jun 19;16(12):2266. doi: 10.3390/cancers16122266 (PMC11202301; doi:10.3390/cancers16122266)
Supplement: Supplementary file 1 [file cancers-16-02266-s001.zip › cancers-3022968-supplementary.pdf]

Supplementary Table S1: Individualized search terms.

**Search key number 1**	(pancreas) and (lesion) and (prospective) and (FNA) or (needle) or (biopsy) or (22G) or (25G)
**Search key number 2**	(pancreas) and (cancer) and (prospective) and (FNA) or (needle) or (biopsy) or (22G) or (25G)

Supplementary Table S2: Number of malignant neoplasia cases.

**Author**	**Total number of cases**	**Malignant neoplasia**	
		**22G**	**25G**
Silvia Carraraa [26]	144	59/72 (81,9%)	64/72 (88,9%)
Peter Vilmann [27]	135	39/62 (62,9%)	42/73 (57,5)
Jun Kyu Lee [28]	188	58/94 (61,7%)	63/94 (67%)
Uzma D. Siddiqui [25]	131	54/64 (84,4%)	61/67 (91%)
Carlo Fabbri [36]	50	34/50 (68%)	40/50 (80%)
Hiroo Imazu [37]	43	NA	NA
Jeffrey H. Lee [33]	12	NA	NA
Georgios Mavrogenis [32]	28	92,9% - no data regarding differences between needles	
Se Woo Park [34]	56	51/56 (91%)	49/56 (87,5%)
Young Sik Woo [29]	206	97/103 (94,2%)	93/103 (90,3%)
Takeshi Tomoda [30]	88	42/43 (97,7%)	42/45 (93,3%)
Antonio Gimeno-Garcia [35]	120	NA	NA
Dongwook Oh [31]	140	59/70 (84,3%)	58/70 (82,9%)
Hiroki Sakamoto [21]	24	NA	NA
